# Decreased ventricular systolic function in chemotherapy-naive patients with acute myeloid leukemia: a three-dimensional speckle-tracking echocardiography study

**DOI:** 10.3389/fcvm.2023.1140234

**Published:** 2023-06-07

**Authors:** Yichan Zhang, Yuting Tan, Tianshu Liu, Yanan Fu, Yixia Lin, Jiawei Shi, Yanting Zhang, Wenhui Deng, Shukun He, Yali Yang, Qing Lv, Li Zhang, Mingxing Xie, Jing Wang

**Affiliations:** ^1^ Department of Ultrasound Medicine, Union Hospital, Tongji Medical College, Huazhong University of Science and Technology, Wuhan, China; ^2^ Clinical Research Center for Medical Imaging in Hubei Province, Wuhan, China; ^3^ Hubei Province Key Laboratory of Molecular Imaging, Wuhan, China

**Keywords:** acute myeloid leukemia, three-dimensional speckle-tracking strain, ventricular systolic function, oncology cardiology, echocardiography

## Abstract

**Background:**

The relationship between acute myeloid leukemia (AML) or acute lymphoblastic leukemia (ALL) and cardiac function is not well established. This study aimed to evaluate whether AML patients exist early myocardial damages prior to chemotherapy and to investigate its association with cardiovascular biomarkers.

**Methods:**

Conventional echocardiography and three-dimensional speckle-tracking strain analysis were performed prospectively in 72 acute leukemia (AL) patients before any chemotherapy therapy (of whom 44 were AML patients, 28 ALL patients). The results were compared with those from 58 control group matched for age and gender.

**Results:**

There were no significant differences in conventional biventricular systolic function parameters between AL patients and controls. The left ventricular global longitudinal strain (LVGLS) and right ventricular free wall longitudinal strain (RVFWLS) were significantly lower in AL patients (−23.0 ± 1.4% vs. −24.1 ± 1.3% and −27.9 ± 7.1% vs. −33.0 ± 4.6%, respectively, *P* < 0.001 for all). Compared with ALL patients, AML patients had lower LVGLS and RVFWLS (−22.7 ± 1.3% vs. −23.5 ± 1.6% and −26.2 ± 7.6% vs. −30.4 ± 5.5%, respectively, *P* < 0.05 for all). LVGLS was lower in ALL patients compared with controls (−23.5 ± 1.6% vs. −24.7 ± 1.4%, *P* < 0.05), however, there was no difference in right ventricular systolic function parameters between the two groups. LVGLS in AL patients was independently correlated with left ventricular ejection fraction (LVEF) and the absolute number of circulating lymphocytes.

**Conclusions:**

Our findings suggest that baseline myocardial systolic function is lower in AL patients than controls. AML patients had lower baseline LVGLS and RVFWLS than controls and ALL patients. The decreased LVGLS is correlated with LVEF and the absolute number of circulating lymphocytes.

## 1. Introduction

For decades, anticancer therapies, such as anthracyclines, cyclophosphamide and trastuzumab, have found broad applications in hematological malignancies and solid tumors, and greatly benefited many patients. However, cardiac injury is the major adverse effect of anticancer treatment (1–3). Many cancer survivors experience not only congestive heart failure, but also myocardial ischemia, thromboembolism, hypertension and arrhythmias that affect their quality of life (1, 4). In a large retrospective study, 3.5% cancer patients developed cardiac toxicity during 10-year period (5). It is worth noting that, higher rates of cardiotoxicity after anthracycline chemotherapy (13%) have been reported in patients with acute leukemia (AL) compared with other types of cancer (6, 7). Patients with acute myeloid leukemia (AML) had an increased incidence of heart failure after anthracycline than patients with acute lymphoblastic leukemia (ALL) (8, 9). After adjustment for age and cumulative anthracycline dose, the presence of AML was still associated with symptomatic HF (9).

AL *per se* may be associated with cardiac abnormalities due to a robust systemic inflammatory response and direct myocardial infiltration by leukemic cells before chemotherapy (10–12). Furthermore, AML and ALL patients may also have different myocardial systolic patterns due to their differences in pathophysiology (13). Current cardiotoxicity studies are mainly focus on the changes in cardiac function during and after chemotherapy (14–17). However, baseline myocardial function in tumor patients is associated with symptomatic heart failure and cardiac death in patients treated with anthracycline (6). A baseline risk assessment is mandatory in all AML patients before initiation of therapy, focusing on early, preclinical detection of cardiotoxicity, providing a basis for early clinical identification of patients with high risk of heart failure and guiding cardioprotective medications. Few studies have been performed in this area.

Abnormalities in strain are reported to occur prior to changes in conventional echo parameters, likely left ventricular ejection fraction (LVEF) in different clinical settings (18, 19). Three-dimensional speckle-tracking echocardiography (3D-STE) is an advanced imaging technique with capabilities to track out-of-plane motion of speckles, higher feasibility and reproducibility over 2D-STE (20, 21). Accordingly, this prospective study was designed to evaluate the application of 3D-STE in identifying subclinical myocardial damages before initiation of chemotherapy.

## 2. Methods

### 2.1. Study population

We prospectively enrolled consecutive AL patients (≥18 years of age) who were referred to Wuhan Union Hospital before any chemotherapy between March 2021 and April 2022. Patients with reduced LVEF (<52% for male and <53% for female) (22), more than mild valvular heart disease, atrial fibrillation, congenital heart disease, previous chemotherapy history and inadequate echocardiographic images were excluded from this study. The AL cohort consisted of a total of 72 patients, of whom 44 were diagnosed with AML and 28 with ALL. All of them had an echocardiogram performed before initiation of chemotherapy. 72 AL patients and 58 cases of control group were matched for age and gender.

This study was approved by the Ethics Committee of Tongji Medical College, Huazhong University of Science and Technology. Written informed consent was obtained from all patients.

### 2.2. Echocardiographic image acquisition

Transthoracic echocardiographic examinations were performed by an experienced echocardiographic doctor using Philip EPIQ 7C (Philips Medical Systems, Andover, USA) equipped with S5-1 and X5-1. All measurements were performed according to the current recommendations of the American Society of Echocardiography (23). 2D echocardiographic acquisitions were recorded from the basal, middle and apical levels of parasternal short axis views and the apical 4-chamber (Ch), 2-Ch and 3-Ch views with 4 consecutive cardiac cycles. The frame rate was set between 55 and 90 frames per second. LVEF was calculated by biplane Simpson's method from apical 4-Ch and 2-Ch views (24). Tricuspid annular plane systolic excursion (TAPSE) was measured as the systolic displacement of the tricuspid lateral annulus, recorded on M-mode imaging. Right ventricular fractional area change (RVFAC) was calculated as: (RV end-diastolic area−RV end-systolic area)/end-diastolic area × 100%. Tricuspid lateral annular systolic velocity (S') was assessed using tissue Doppler imaging from the apical 4-ch view.

3D echocardiographic acquisitions were recorded from the four-chamber apical view in heart model mode with volume rate of 19–25 volumes/s, and were gathered over four cardiac cycles, during a breath-hold lasting for a few seconds. Left ventricular end-systolic volume (LVESV), end-diastolic volume (LVEDV), right ventricular end-systolic volume (RVESV), end-diastolic volume (RVEDV) and RV ejection fraction (RVEF) were measured by 3D echocardiography. The 2D and 3D echocardiographic datasets were stored digitally for offline analysis.

### 2.3. 3D strain analysis

**Figure 1:**
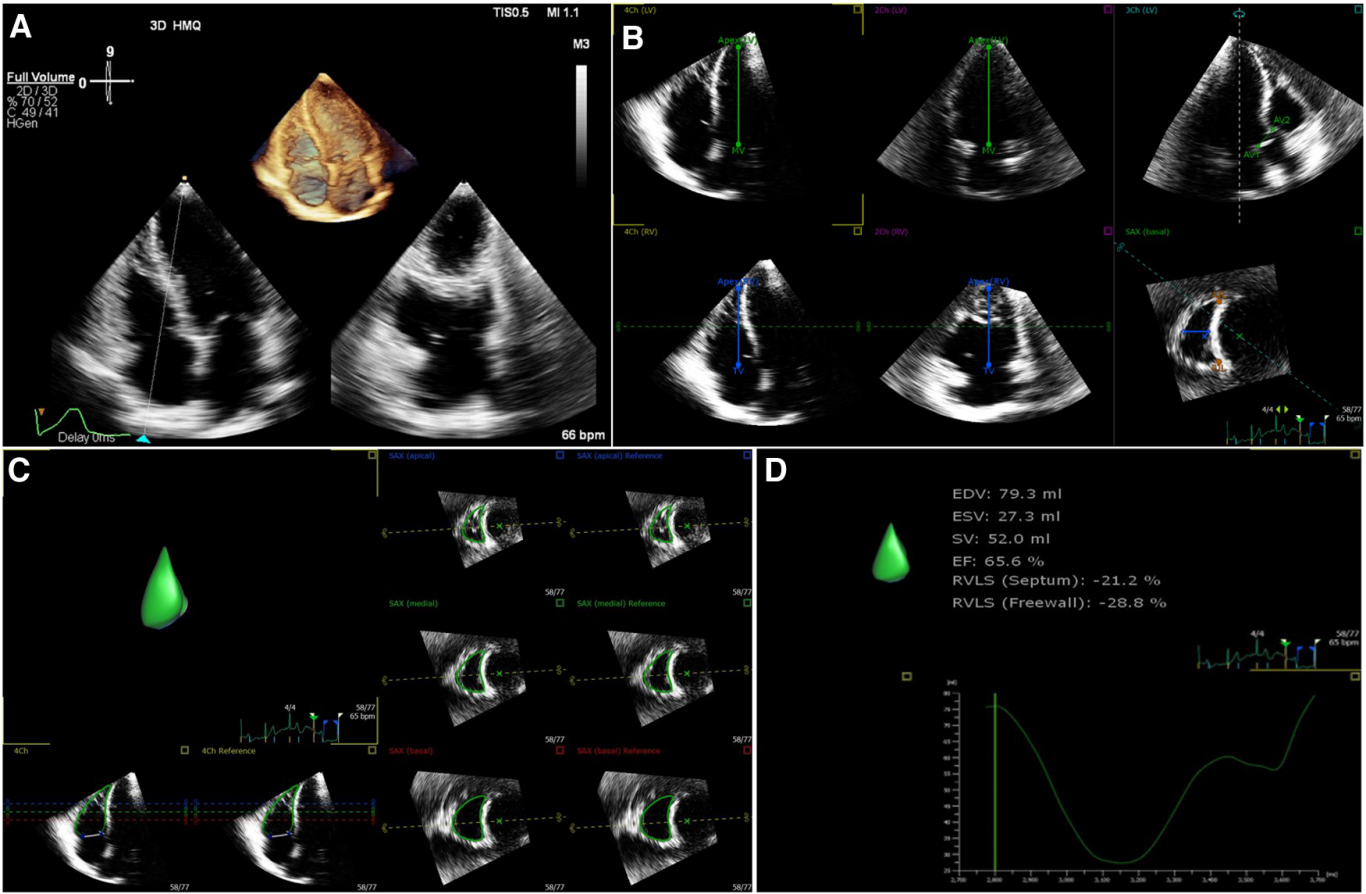
Rv three-dimensional image. (**A**) Three-dimensional image of RV-focused apical 4-chamber view. (**B**) Reference points setting. (**C**) RV endocardial border identification and tracking at end-systole and end-diastole. (**D**) Longitudinal strain of RV free wall and septum. RV, right ventricular.

The off-line 3D-STE analyses were performed using the vendor-independent software TomTec (4D LV-Analysis 3.1 for 3D-STE Tom Tec Imaging Systems, Unterschleissheim, Germany) by an experienced researcher who was blinded to the groups. The software semi-automatically aligned and displayed the LV in the short axis view at level of the aortic valve and 3 standard apical views (25). To avoid foreshortening, manual adjustment was necessary to obtain the maximal LV long axis view. The endocardial surface of the ventricle in end-diastolic and end-systolic frames was identified automatically for initial contour detection. The operator could manually adjust the ventricular endocardial surface if necessary. Subsequently, the software automatically tracked the endocardium throughout the entire cardiac cycle, then the software provides the time-volume and time-strain curves (Figure 1, Supplementary Figure S1).

The 3D LV global longitudinal strain (LVGLS) and global circumferential strain (LVGCS) were quantified as the mean of the strain values from all 16 segments of LV. The 3D RV free wall longitudinal strain (RVFWLS), RV septal wall longitudinal strain (RVSWLS) and RV global longitudinal strain (RVGLS) were acquired simultaneously. The patients with persistent inadequate tracking were excluded from the study.

### 2.4. Reproducibility analysis

Intraobserver and interobserver variability of the 3D-STE parameters were analyzed in 20 randomly selected patients from AL group. Intraobserver reproducibility was assessed by the same observer performed 4 weeks later again. Interobserver reproducibility was assessed by the second observer who was blinded to the measurements of the first observer in the same 20 patients.

### 2.5. Statistical analysis

Continuous variables are presented as mean ± standard deviation or as median (interquartile range), and categorical variables as number (%). Comparisons of clinical and echocardiographic parameters between AL patients and control group were performed using the Student's *t* test or the Wilcoxon rank sum tests for continuous variables and the *χ*^2^ or Fisher's exact test for categorical variables. Comparisons of echocardiographic parameters between AML and ALL patients were performed using one-way analysis of covariance. Correlations between LVGLS and clinical, laboratory, and echocardiographic variables were estimated using Pearson correlation coefficients or Spearman *ρ* test for nonparametric values. A multivariable linear regression was also performed to explore the independent clinical and echocardiographic parameters associated with GLS in patients with AL. All variables reaching *P* values of < 0.1 on univariate analysis were included in the multivariate analyses. The intraobserver and interobserver variability of 3D-STE parameters were assessed by the intraclass correlation coefficients (ICCs) and the Bland–Altman analyses in a random sample of 20 patients. All statistical analyses were performed on SPSS version 26.0 (Statistical Package for the Social Sciences, Chicago, Illinois) and GraphPad Prism version 8.0.1. A 2-sided *P* value of < 0.05 was considered as statistically significant.

## 3. Results

### 3.1. Study population

There was no difference in age, sex distribution, BMI, diabetes, prevalence of hypertension, dyslipidemia, smoking, prior coronary artery disease (CAD) and chronic kidney disease (CKD) between AL patients and controls. The history of medications did not differ between the two groups (Table 1).

**Table 1:** Clinical characteristics of patients with AL and control group.

Variable	Total (*n* = 130)	AL (*n* = 72)	Control group (*n* = 58)	*P* value
Age, years	49 (37–58)	51 (39–59)	47 (33–58)	0.234
Female sex	56 (43)	31 (43)	25 (43)	0.217
BMI, kg/m^2^	22.9 ± 2.8	22.8 ± 2.9	23.0 ± 2.6	0.684
**Cardiac risk factors**				
Diabetes	10 (8)	6 (8)	4 (7)	≥0.999
Hypertension	27 (21)	18 (25)	9 (16)	0.185
Dyslipidemia	20 (15)	12 (17)	8 (14)	0.652
Current smoking	16 (12)	10 (14)	6 (10)	0.541
Prior CAD	6 (5)	4 (6)	2 (3)	0.691
Prior CKD	10 (8)	7 (10)	3 (5)	0.524
**Medications**				
β-blockers	7 (5)	4 (6)	3 (5)	≥0.999
ACE inhibitors/ARBs	4 (3)	3 (4)	1 (2)	0.628
Statins	6 (5)	2 (3)	4 (7)	0.406

Data are expressed as mean ± SD, as median (interquartile range), or as number (percentage). BMI, body mass index; CAD, coronary artery disease; CKD, chronic kidney disease; ACE, angiotensin-converting enzyme; ARB, angiotensin receptor blocker.

### 3.2. AL patients vs. control group

LV volume and LV volume indexed to body surface area were slightly higher in patients with AL (*P* < 0.05). Systolic dyssynchrony index (SDI) was similar between the two groups. Although LVEF was similar between AL patients and controls (61.1 ± 4.2% vs. 62.4 ± 4.9%, *P* > 0.05), a statistically significant difference was noted with regard to LVGLS (−23.0 ± 1.4% vs. −24.1 ± 1.3%, *P* < 0.001). There was no significant difference in LVGCS (Table 2).

**Table 2:** Echocardiographic parameters of patients with AL and control group.

Variable	Total (*n* = 130)	AL (*n* = 72)	Control Group (*n* = 58)	*P* value
**Left ventricle**				
LVEDV, ml	87.1 ± 17.9	90.9 ± 17.9	84.5 ± 17.0	0.007
LVEDVI, ml/m^2^	48.6 ± 9.4	50.8 ± 9.7	45.9 ± 8.5	0.003
LVESV, ml	33.5 ± 8.3	35.4 ± 8.6	31.0 ± 7.3	0.002
LVESVI, ml/m^2^	18.6 ± 4.4	19.7 ± 4.5	17.2 ± 3.7	0.001
LVSV, ml	53.7 ± 11.4	55.5 ± 11.0	51.5 ± 11.5	0.046
LVSVI, ml/m^2^	30.0 ± 6.1	31.0 ± 6.1	28.6 ± 5.9	0.026
LVEF, %	61.7 ± 4.5	61.2 ± 4.2	62.4 ± 4.9	0.124
SDI, %	6.2 ± 1.7	6.3 ± 1.7	6.0 ± 1.7	0.226
3DGLS, %	−23.2 ± 1.4	−23.0 ± 1.4	−24.1 ± 1.3	<0.001
3DGCS, %	−29.5 ± 4.0	−28.9 ± 3.3	−30.3 ± 4.6	0.053
**Right ventricle**				
FAC, %	49.9 ± 5.1	48.3 ± 5.1	49.9 ± 4.9	0.085
TAPSE, cm	2.2 ± 0.3	2.1 ± 0.3	2.2 ± 0.3	0.394
S’, cm/s	14.2 ± 2.1	14.3 ± 2.2	14.2 ± 2.1	0.932
RVEDV, ml	61.4 ± 15.4	62.5 ± 14.1	60.7 ± 16.8	0.494
RVEDVI, ml/m^2^	34.1 ± 7.5	34.8 ± 6.9	33.6 ± 8.1	0.360
RVESV, ml	24.4 ± 6.7	25.0 ± 6.7	23.8 ± 6.6	0.303
RVESVI, ml/m^2^	13.5 ± 3.2	13.9 ± 3.3	13.1 ± 3.1	0.188
RVSV, ml	37.0 ± 10.2	37.5 ± 9.3	36.9 ± 11.2	0.721
RVSVI, ml/m^2^	20.6 ± 5.2	20.9 ± 4.9	20.4 ± 5.6	0.611
3DRVEF, %	60.2 ± 5.1	60.0 ± 5.7	60.6 ± 4.5	0.507
3DRVFWLS, %	−30.1 ± 6.6	−27.9 ± 7.1	−33.0 ± 4.6	<0.001
3DRVSWLS, %	−23.8 ± 6.0	−23.8 ± 6.3	−23.4 ± 5.6	0.703
3DRVGLS, %	−27.0 ± 4.6	−25.9 ± 4.7	−28.2 ± 4.3	0.004

Data are expressed as mean ± SD. EDV, end-diastolic volume; EDVI, end–diastolic volume index; ESV, end-systolic volume; ESVI, end-systolic volume index; SV, stroke volume; SVI, stroke volume index; SDI, systolic dyssynchrony index; GLS, global longitudinal strain; GCS, global circumferential strain; FAC, fractional area change; TAPSE, tricuspid annular plane systolic excursion; S’, tricuspid annular systolic excursion velocity; RVFWLS, RV free wall longitudinal strain; RVSWLS, RV septal wall longitudinal strain.

Conventional parameters of RV systolic function (FAC, TAPSE and S’) were similar between two groups. Compared with controls, the RVGLS and RVFWLS were lower in AL patients (−25.9 ± 4.7% vs. −28.2 ± 4.3%, *P* < 0.05 and −27.9 ± 7.1% vs. −33.0 ± 4.6%, *P* < 0.001, respectively) (Table 2). There was no significant difference in RVSWLS.

### 3.3. AML patients vs. ALL patients

**Figure 2:**
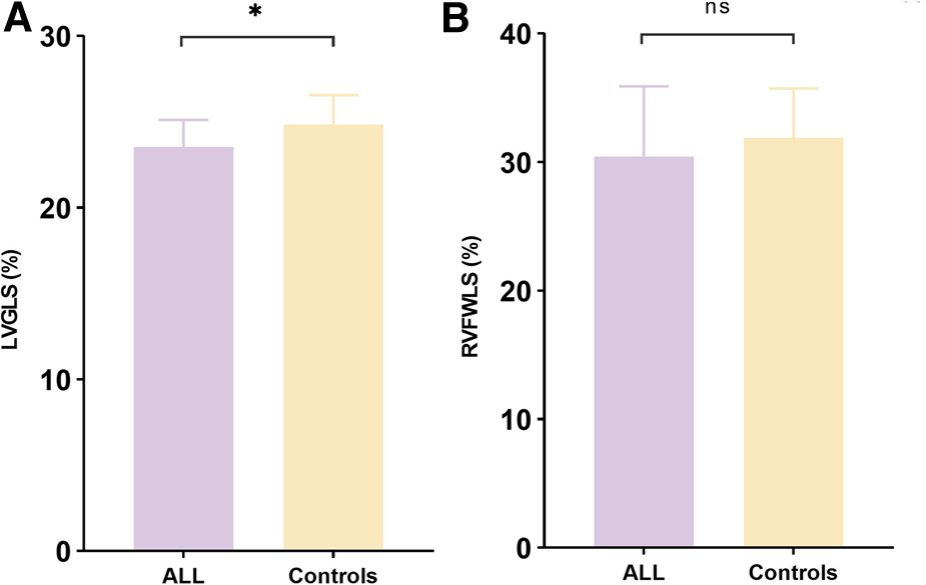
LVGLS and RVFWLS in patients with ALL (*n* = 28) and controls (*n* = 20). (**A**) Comparison of LVGLS between patients with ALL and controls. (**B**) Comparison of RVFWLS between patients with ALL and controls. LVGLS, left ventricle global longitudinal strain; RVFWLS, right ventricular free wall longitudinal strain; ALL, acute lymphoblastic leukemia; LVGLS and RVFWLS values are absolute values; *P < 0.05 compared with controls; ns, not statistically different.

After adjustment for hemoglobin counts, only LVGLS and RVFWLS were lower in patients with AML (−22.7 ± 1.3% vs. −23.5 ± 1.6% and −26.2 ± 7.6% vs. −30.4 ± 5.5%, respectively, *P* < 0.05 for all) (Table 3). LVGLS was lower in ALL patients compared with controls (−23.5 ± 1.6% vs. −24.7 ± 1.4%, *P* < 0.05). However, there was no difference in RV systolic function parameters between the two groups (Figure 2).

**Table 3:** Echocardiographic parameters in AML and ALL patients after adjustment for hemoglobin.

	AML (*n* = 44)	ALL (*n* = 28)	*P* value	
			Unadjusted	Adjusted
LVEF, %	60.7 ± 3.7	61.9 ± 4.9	0.318	0.169
3DGLS, %	−22.7 ± 1.3	−23.5 ± 1.6	0.022	0.005
3DGCS, %	−28.5 ± 3.6	−29.5 ± 2.8	0.205	0.210
3DRVEF, %	59.4 ± 5.5	60.9 ± 5.8	0.396	0.140
3DRVFWLS, %	−26.2 ± 7.6	−30.4 ± 5.5	0.014	0.004
3DRVSWLS, %	−24.9 ± 6.6	−22.2 ± 5.5	0.081	0.101
3DRVGLS, %	−25.6 ± 4.7	−26.3 ± 4.8	0.507	0.298

Data are expressed as mean ± SD.

### 3.4. Clinical, laboratory, and echocardiographic parameters associated with GLS in AL patients

**Figure 3:**
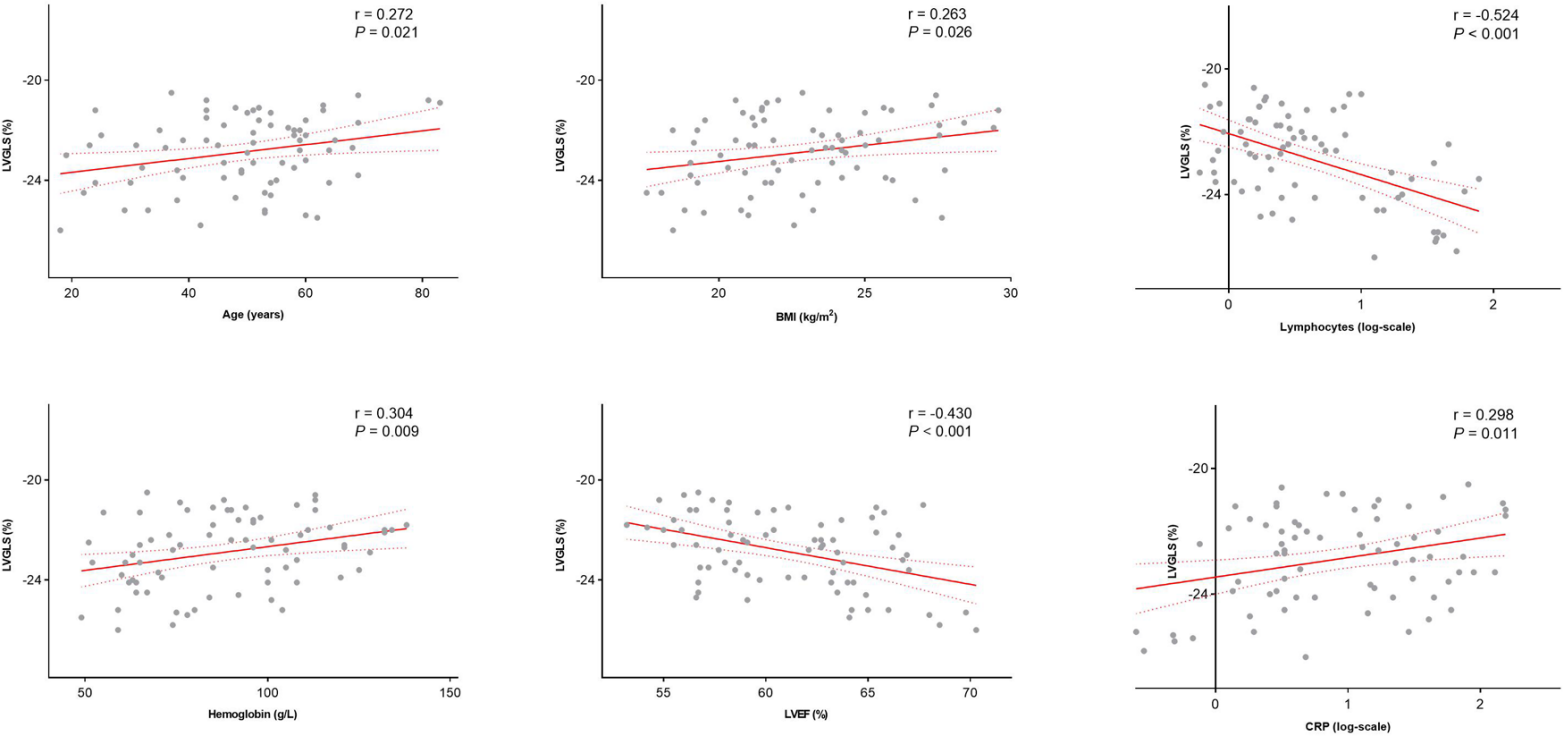
Correlation between LVGLS and age, BMI, absolute number of lymphocytes, hemoglobin counts, LVEF and absolute number of CRP among AL patient. LVEF, left ventricular ejection fraction; CRP, C reactive protein.

Among all AL patients, LVGLS was correlated with LVEF (r = −0.430, *P* < 0.001), BMI (r = 0.263, *P* = 0.026), age, (r = 0.272, *P* = 0.021), the absolute number of C reactive protein (CRP) (r = 0.298, *P* = 0.011), the absolute number of circulating lymphocytes (r = −0.524, *P* < 0.001) and hemoglobin counts (r = 0.304, *P* = 0.009; Figure 3). Therefore, a decrease in LVGLS was associated with a reduction in the absolute number of lymphocytes and LVEF and with an increase in age, BMI, the absolute number of CRP and hemoglobin counts. In a multivariable linear regression analysis, the factors independently associated with LVGLS were LVEF (β = −0.085, *P* = 0.019) and the absolute number of circulating lymphocytes (β = −0.814, *P* = 0.004). However, RVFWLS was only correlated with BMI (r = 0.323, *P* = 0.005) and hemoglobin counts (r = 0.267, *P* = 0.021) (Supplementary Table S1).

### 3.5. Reproducibility of 3D strain measurements

Intraobserver and interobserver variability of the 3D-STE parameters are presented in Table 4. Strain parameters measured by 3D-STE showed excellent reproducibility, as evidenced by the high ICC, small bias, and narrow limits of agreement.

**Table 4:** Intraobserver and interobserver reproducibility for 3D-STE parameters.

	ICC	95% CI	Bias	Limits of agreement
Intraobserver				
LVGLS	0.918	0.807–0.967	−0.11	(−1.698, 1.488)
LVGCS	0.811	0.569–0.922	0.695	(−3.673, 2.283)
RVFWLS	0.895	0.757–0.957	0.695	(−3.652, 5.042)
RVSWLS	0.872	0.673–0.950	−1.08	(−5.216, 3.056)
Interobserver				
LVGLS	0.918	0.806–0.967	−0.04	(−1.713, 1.633)
LVGCS	0.912	0.793–0.964	−0.695	(−2.633, 2.383)
RVFWLS	0.889	0.742–0.955	0.21	(−4.223, 4.643)
RVSWLS	0.921	0.814–0.968	−0.41	(−4.105, 3.275)

## 4. Discussion

To the best of our knowledge, this is the first study to evaluate the myocardial systolic function before chemotherapy in AML and ALL patients by 3D-STE. The present investigation revealed several interesting findings that deserve comment. (1) AL patients had lower baseline LVGLS and RVGLS. (2) AML patients had lower baseline LVGLS and RVFWLS. (3) Among AL patients, LVGLS was independently associated with LVEF and the absolute number of circulating lymphocytes.

The majority of studies have focused on LV dysfunction induced by different chemotherapeutic agents — anthracyclines and trastuzumab in patients with cancer. Previous studies (26, 27) have showed that AL by itself may be associated with cardiac alterations. Several factors may explain the prechemotherapy myocardial remodeling in AL patients. It had been confirmed that the imbalance between pro-inflammatory and anti-inflammatory cytokines is an important reason for the formation of leukemia blasts. Elevated plasma CRP levels were found in most AL patients (28). The activation of inflammation in the heart causes LV remodeling and dysfunction and is associated with adverse outcomes in patients with heart failure. Another possible mechanism to explain cardiac alterations in patients with AL is direct infiltration of the heart by leukemic cells, which has been extensively described in previous autopsy studies (10, 29).

Our research demonstrates that LVGLS is attenuated in AML patients compared with ALL patients. Plasma TNF-a, interleukin-6 (IL-6) and IL-10 levels were higher in AML patients compared with ALL patients and control group and plasma levels of IL-6 and IL-10 were associated with patient survival and event-free survival. A significantly higher incidence of cardiac infiltration has been found in AML patients than in ALL patients revealed by autopsy findings (10), which may be the reason for lower LVGLS in AML patients (11, 12). Some evidence has shown an association between somatic mutations in AML patients and an increased risk for coronary artery disease, thus being a potential mechanism pathway (30). Furthermore, mutations in isocitrate dehydrogenase 1 and 2, with an overall frequency of approximately 9% to 16% in patients with AML, have been implicated in cardiac dysfunction (31). This is important, because AML patients, a population with many senior citizens, show increased cardiac-specific mortality (8). Ali et al. (6) showed that prechemotherapy LVGLS is an effective tool to stratify patients at high risk for cardiac events after anthracycline therapy and may help tailor treatments to decrease anthracycline-induced cardiotoxicity. More attention should be paid to cardiac function of AML patients during and after chemotherapy.

RVFWLS was decreased in AML patients compared to ALL patients despite normal RVSWLS and RVGLS, which may reflect early systolic function changes of RV myocardium. Longitudinal RV function is substantial, accounting for >60% of the ejection fraction and might be reflected by RVFWLS (32). Global RV systolic parameters, for example RVGLS, the average strain value obtained by tracking six segments of the RV, may be affected by LV systolic function owing to the fact that the interventricular septum is conventionally regarded as one part of LV (33). RVFWLS may serve as an early marker of altered RV myocardial function. Considering the fact that the reduction of RVFWLS was significantly correlated with dyspnea severity and heart failure, RVFWLS may play an important role in the follow up of AML patients (34).

Our study did not show significant change in echocardiographic parameters of ventricular structure and function between ALL patients and controls except for LVGLS. Baseline myocardial contraction alterations need to be demonstrated in a longer course.

In AL patients, decreased LVGLS was correlated with reduced LVEF and the absolute number of circulating lymphocytes. Cardiac dysfunction represents a serious complication in patients with cancer. Although cardiotoxicity is generally related to chemotherapy and other anticancer therapies, the impact, molecular mechanisms and biological basis of the effects induced by tumor growth on cardiac functions, regardless of therapy, still remain unclear and little investigated (12). Previous studies have shown that the systemic inflammatory response may result in cardiac damage (35). In our study, the increased number of CRP and decreased number of circulating lymphocytes were associated with LVGLS reduction. We speculate that cancer growth could exert *per se*, independently from chemotherapy effects, cardiotoxicity, and inflammatory molecules could be responsible for promoting LV systolic dysfunction (36).

### 4.1. Limitations

This study has several limitations. First, this was a single-center study and the sample size was relatively small. Second, we did not acquire all the laboratory biomarker tests of our patients, such as high-sensitivity troponin I (hs-TNI) and IL-6, which reflect the level of inflammation in the body. Third, our study lacks histological confirmation of interstitial inflammatory infiltrates of myocardial tissue. Fourth, 3D-STE technique was dependent on image quality, and the value of strain in our study may not apply to other software algorithms due to inter-vendor variability. Therefore, future studies with multicenter involvement may strengthen the study power.

## 5. Conclusion

Our findings suggest that baseline myocardial systolic function is lower in AL patients than controls. AML patients had lower baseline LVGLS and RVFWLS than controls and ALL patients. The decreased LVGLS is correlated with LVEF and the absolute number of circulating lymphocytes. These findings suggest that AL by itself maybe associated with cardiac alterations and may help understand the higher cardiovascular risk and cardiotoxicity after chemotherapy in AL patients compared to other types of cancer. Further studies are needed to investigate the pathophysiologic basis of preexisting cardiac alterations.

## Data Availability

The original contributions presented in the study are included in the article/Supplementary Material, further inquiries can be directed to the corresponding author/s.

## Supplementary material

Supplementary Figure LV three-dimensional image. (A) Three-dimensional image of LV apical 4-chamber view. (B) Reference points setting. (C) LV endocardial border identification and tracking at end-systole and end-diastole. (D) Longitudinal strain of LV. LV, left ventricular.
