# Supplementary material for: Decreased ventricular systolic function in chemotherapy-naive patients with acute myeloid leukemia: a three-dimensional speckle-tracking echocardiography study
Source: Front Cardiovasc Med. 2023 Jun 7;10:1140234. doi: 10.3389/fcvm.2023.1140234 (PMC10282833; doi:10.3389/fcvm.2023.1140234)
Supplement: Supplementary file 1 [file Table1.docx]

**Supplemental Information**

| Table 1 Multivariable linear regression analysis of LVGLS in AL patients | | |
| --- | --- | --- |
| Variable | β（95% CI） | *P* value |
| LVEF, % | -0.085（-0.156, -0.015） | 0.019 |
| Age, years | 0.007（-0.014, 0.027） | 0.527 |
| BMI, kg/m^2^ | 0.061（-0.042, 0.164） | 0.239 |
| Hemoglobin, G/L | 0.006（-0.007, 0.02） | 0.35 |
| Lymphocyte, G/L | -0.814（-1.365, -0.263） | 0.004 |
| CRP, mg/L | 0.382（-0.035, 0.799） | 0.072 |
|  | | |
