# Supplementary figures and images for: Decreased ventricular systolic function in chemotherapy-naive patients with acute myeloid leukemia: a three-dimensional speckle-tracking echocardiography study
Source: Front Cardiovasc Med. 2023 Jun 7;10:1140234. doi: 10.3389/fcvm.2023.1140234 (PMC10282833; doi:10.3389/fcvm.2023.1140234)

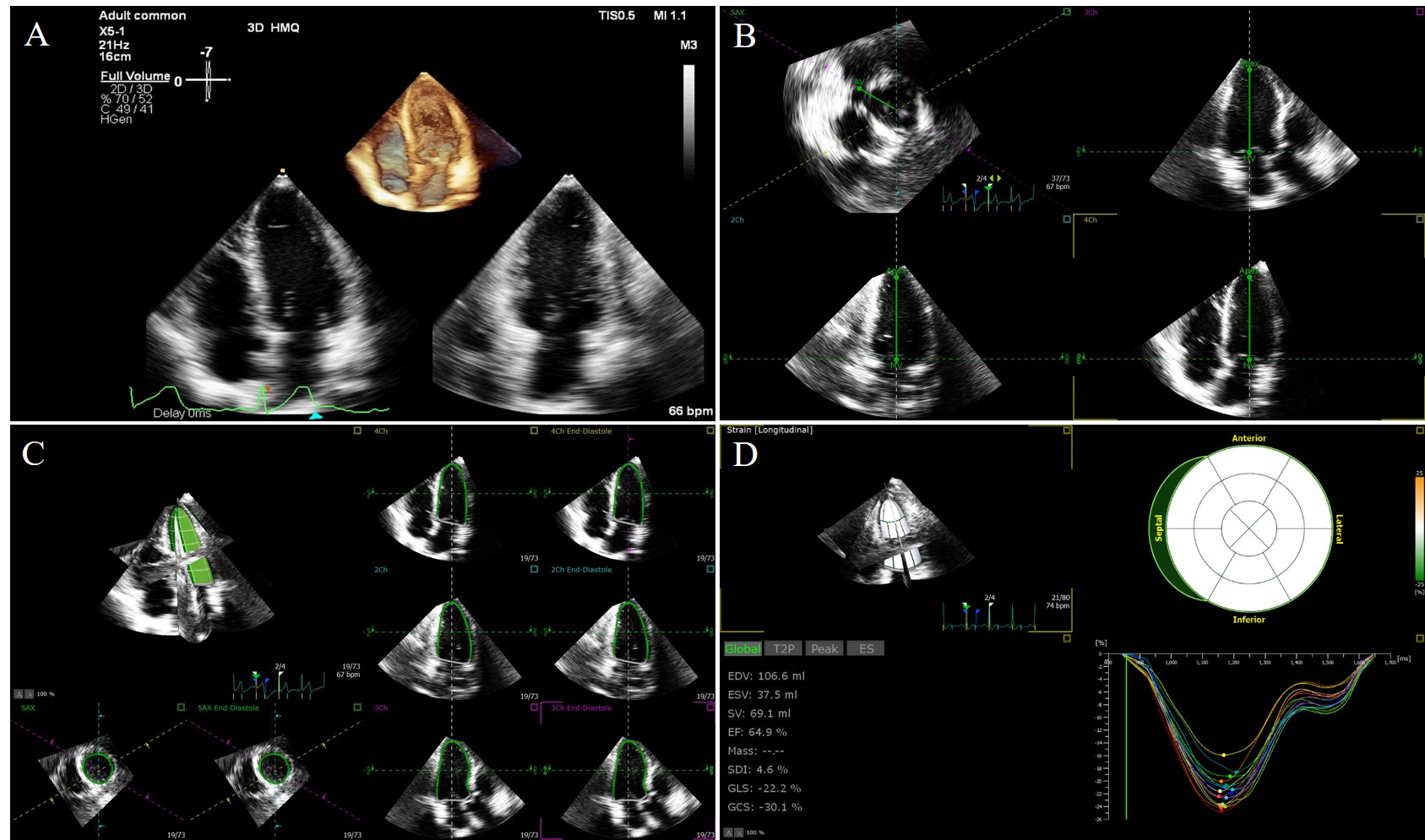

Supplement: Supplementary Figure — LV three-dimensional image. (A) Three-dimensional image of LV apical 4-chamber view. (B) Reference points setting. (C) LV endocardial border identification and tracking at end-systole and end-diastole. (D) Longitudinal strain of LV. LV, left ventricular. [file Image1.jpeg]
